# Effects of Chronic D-Serine Elevation on Animal Models of Depression and Anxiety-Related Behavior

**DOI:** 10.1371/journal.pone.0067131

**Published:** 2013-06-21

**Authors:** David-Marian Otte, Maria Luisa Barcena de Arellano, Andras Bilkei-Gorzo, Önder Albayram, Sophie Imbeault, Haang Jeung, Judith Alferink, Andreas Zimmer

**Affiliations:** 1 Institute of Molecular Psychiatry, University of Bonn, Bonn, Germany; 2 Department of Psychiatry, University of Bonn, Bonn, Germany; McLean Hospital/Harvard Medical School, United States of America

## Abstract

NMDA receptors are activated after binding of the agonist glutamate to the NR2 subunit along with a co-agonist, either L-glycine or D-serine, to the NR1 subunit. There is substantial evidence to suggest that D-serine is the most relevant co-agonist in forebrain regions and that alterations in D-serine levels contribute to psychiatric disorders. D-serine is produced through isomerization of L-serine by serine racemase (Srr), either in neurons or in astrocytes. It is released by astrocytes by an activity-dependent mechanism involving secretory vesicles. In the present study we generated transgenic mice (SrrTg) expressing serine racemase under a human GFAP promoter. These mice were biochemically and behaviorally analyzed using paradigms of anxiety, depression and cognition. Furthermore, we investigated the behavioral effects of long-term administration of D-serine added to the drinking water. Elevated brain D-serine levels in SrrTg mice resulted in specific behavioral phenotypes in the forced swim, novelty suppression of feeding and olfactory bulbectomy paradigms that are indicative of a reduced proneness towards depression-related behavior. Chronic dietary D-serine supplement mimics the depression-related behavioral phenotype observed in SrrTg mice. Our results suggest that D-serine supplementation may improve mood disorders.

## Introduction

The ionotropic N-methyl-D-aspartic acid (NMDA) receptor is activated by glutamate [1], if an allosteric co-activation site is occupied by either glycine or D-serine [2]. While glycine is probably the main co-agonist in the spinal cord and in the hindbrain, several lines of evidence indicate that D-serine is the main co-agonist in the forebrain [3]–[8]. Thus, glycine concentrations at forebrain glutamatergic synapses are low, most probably due to the presence of high-affinity glycine transporters in closely apposed astrocytes [9]–[14]. In contrast, the forebrain contains relatively high levels of D-serine [6], [7], [15], [16], particularly in regions with high NMDA receptor expression [6], [7], [16]. For instance, D-serine is found at high concentrations in hippocampal astrocytes located in close proximity to CA1 apical dendrites, which contain a high NMDA receptor density [6]. D-serine is also a more potent activator of NMDA receptors than glycine when examined in heterologous expression systems [17], [18], or in brain slices [9]. Recent studies demonstrated that the NMDA receptor glycine/D-serine site is most probably not fully saturated in many brain regions (i.e. hippocampus, thalamus, neocortex, brain stem and retina) [9], [10], [13], [14], [19]–[22]. Fine-tuning of D-serine levels may therefore serve as a mechanism to modulate NMDA receptor activity. Most recently, it has been shown that synaptic and extrasynaptic NMDARs are gated by D-serine and glycine, respectively. The regionalized availability of the coagonists matches the favored affinity of synaptic NMDARs for D-serine and extrasynaptic NMDARs for glycine. Interestingly, Papouin et al. demonstrated that long-term potentiation and NMDA-induced neurotoxicity rely on synaptic NMDARs only while long-term depression requires both [23]. In the mammalian brain, D-serine is produced from L-serine by the enzyme serine racemase [3], [24]–[26]. Neurons are probably the main source of serine racemase and D-serine in mammals, because serine racemase is predominantly localized in this cell type in the brain. Serine racemase has also been found to a lesser extent in astrocytes [27]). Interestingly, despite being less expressed, astrocytic D-serine seems to be essential for hippocampal LTP [28].

Alterations in glutamatergic signaling through NMDA receptors have been implicated in the pathology of psychiatric disorders and thus a tight regulation of D-serine levels may be important [29], [30]. Indeed, it has been shown that patients with mood disorders display increased post mortem brain glutamate levels [31] and schizophrenic patients exhibit reduced D-serine levels in the cerebrospinal fluid (CSF) [32].

Glycine site agonists were found to be beneficial in several chronic neurological disorders, including Alzheimer's disease and schizophrenia [33]–[36]. Additionally, D-cycloserine, a partial agonist at the strychnine-insensitive glycine site of the NMDA-receptor [37], facilitates declarative learning and hippocampal activity in healthy humans [38]. A role of D-serine in the modulation of affective behaviors was recently demonstrated using a serine racemase knockout mouse model with dramatically reduced D-serine levels. These mice exhibited elevated levels of anxiety [39] and a decline of cognitive functions [39]. Furthermore, it has been shown that an acute treatment with D-serine produces antidepressant effects in rodents [40].

We have now generated a mouse strain with chronically increased D-serine levels by transgenic expression of serine racemase in astrocytes (SrrTg). These animals were analyzed in behavioral paradigms of depression and cognition to determine the behavioral effects of increased D-serine levels. SrrTg mice showed a reduced depression-related behavior in the ultrasonic vocalization, the forced swim and the novelty-suppressed feeding test. Higher brain serine racemase expression did not alter the animals' learning ability, as evidenced by the results of the operant learning paradigm and the water maze test. Interestingly, D-serine added to the drinking water in wild type (Wt) mice produced behavioral changes that were similar to the SrrTG phenotype.

## Materials and Methods

### Generation of transgenic mice

**Figure 1:**
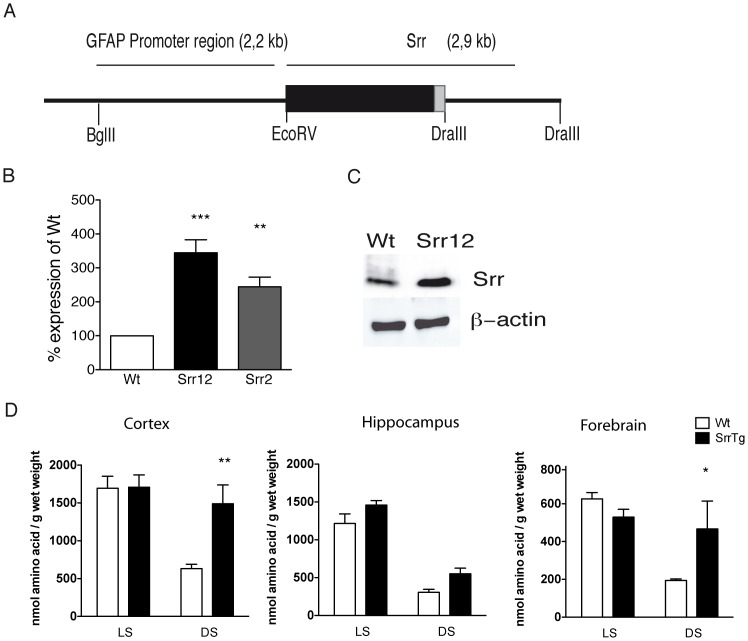
Serine racemase expression and brain D-serine levels in transgenic mice. (A) Schematic representation of the construct for serine racemase overexpression in glial cells under a GFAP promoter. The GFAP promoter is represented as a black line, the serine racemase ORF as a black bar and the SV40 intron/polyA signal as a grey bar. The cutting sites for the endonucleases *Bgl*II, *Eco*RV and *Dra*III are listed. (B) Quantitative analysis of serine racemase expression in adult mouse brains by TaqMan® Assay. Serine racemase expression was calculated as ΔC_T_ value, normalized to GAPDH. Shown are the average values of 4 animals, expressed as mean ±SEM. (C) Western blot analysis of serine racemase in whole brain of a Srr12 mouse and a Wt littermate. Images of *Western* blot analysis of serine racemase and β-actin were performed with specific antibodies using the same extract (n = 4). All values are expressed as mean ±SEM of 5 animals; *p<0.05 and **p<0.01. (D) Quantitative analysis of absolute brain D-serine and L-serine levels per wet weight in 5 Srr12 mice and corresponding Wt littermates by HPLC. D-serine levels of cortex, hippocampus and forebrain were measured. Mice were treated with D-serine (D), L-serine (L), glycine (G) and water (W).

For the generation of the expression construct, we obtained a vector containing the complete murine serine racemase ORF (BC011164-NCBI) under the control of the CMV promoter (CMV-SPORT6 backbone; RZPD, Germany, order nr. IRAKp961M0624Q). A 2.4 kb *Dra*III/*Eco*RV fragment harboring the serine racemase ORF, the SV40 intron and polyA were cut out and cloned into a *NotI/BamHI* restricted and blunted pGFAP-GFP vector [41]. The resulting GFAP-serine racemase vector was cut with BglII/DraIII to release a 5.1 kb fragment containing the GFAP promoter, the serine racemase ORF and the SV40 intron and polyA (Fig 1A). This fragment was gel-purified, diluted in oocyte injection buffer (5 mM Tris, pH 7.4, 0.2 mM EDTA, 100 mM NaCl) to 3 ng/µl and injected into pronuclei of fertilized oocytes from CD1 mice. The transgenic founder mice were crossed to CD1 Wt mice. Transgenic lines were maintained and analyzed as hemizygous lines (Srr2 and Srr12) using CD1 Wt mice for breeding. Genotyping was done by PCR with genomic DNA obtained from mouse tail biopsies using the primers SrrTg F (5′TTCGAGGTGCCCTTAATGCC′3) and SrrTg R (5′AGAGCTTGGC CATGGTTTCC′3).

### Total RNA preparation and RT PCR

Mouse brain tissues from Srr12, Srr2 and Wt mice were rapidly dissected, snap frozen in isopentane and stored at −80°C. Total RNA was prepared using the Trizol method (Invitrogen, Germany). Five µg RNA and 0.5 µg oligo dT (20) primers (Invitrogen) were heated at 70°C for 4 min, chilled on ice and reverse transcribed in a total volume of 20 µl containing 4 µl first strand buffer (Invitrogen), 2 µl 0.1 M DTT, 1 µl 10 mM dNTPs, 0.5 µl RNase OUT (Invitrogen), and 200 U Superscript II reverse transcriptase (Invitrogen) at 42°C for 50 min.

### Taqman analysis

Real-time quantitative PCR was performed using an ABI 7900 sequence detector (Perkin Elmer, MA, USA) on cDNA samples. The PCR reaction was carried out at 50°C for 2 min, 95°C for 10 min followed by 40 cycles of 95°C for 15 s, then 60°C for 1 min using the manufacturers Universal PCR Master Mix (Perkin Elmer, MA, USA). Taqman primer and probe sets were purchased from Applied Biosystems: Mm00489125_m1 for serine racemase and for glyceraldehyde-3-phosphate-dehydrogenase (GAPDH). GAPDH was used to normalize for the amount of sample in a given reaction. Results are expressed as fold change calculated relative to CD1 Wt after normalization.

### Western blot analysis

Brains were homogenized in 10 mM Tris HCl pH 8, 150 mM NaCl, 5 mM EDTA, 1% NP-40, 0.5% sodium deoxycholate, and 0.1% SDS containing Complete Mini protease inhibitor cocktail (Roche). Protein concentration was determined using the BCA Protein Assay (Pierce). 20 µg of the protein extracts were separated by 12% SDS-PAGE and probed with mouse anti-serine racemase polyclonal antibody (1∶500; BD, overnight at 4°C) followed by rabbit anti-mouse peroxidase-conjugated antibody (1∶10,000; Jackson ImmunoResearch, 1 h at room temperature) and then exposed to enhanced chemiluminescence substrate (ECL; Pierce) for 5 minutes. After stripping, the blot was labeled with a mouse anti-β-actin antibody (Sigma A2228, 1∶10000, 2 h at room temperature) as a housekeeping gene followed by a goat □nti-mouse peroxidase-conjugated antibody (Jackson Lab, 1∶3000, 1 h at room temperature) and again incubated with ECL for 5 minutes. The blots were analyzed using the ChemiDoc™ MP Imagine System from Bio-Rad and the ImageLab 4.01 software.

### Immunohistochemistry

All procedures were performed at room temperature and all solutions were dissolved in 1X PBS (Gibco 18912-014) unless otherwise indicated. All chemicals were purchased from Sigma-Aldrich unless otherwise specified. Tissues were obtained from 8–9 week old mice transcardially perfused with PBS followed by 4% paraformaldehyde and post-fixed between 6–16 hours at 4°C. Organs were cryoprotected in 20% sucrose and 20 µm-thick slices were cut using a cryostat (Leica CM3050S), mounted onto glass slides (FisherBrand Superfrost Plus) and stored at −80°C until further use.

Sections were thawed for 20 minutes in PBS and permeabilized using 0.2% Triton-X 100 for 20 minutes before they were washed in PBS for 5 minutes. Blocking of unspecific binding sites was done with 5% goat serum (Abcam) for 10 minutes followed by another PBS wash for 5 minutes. Rabbit anti-serine racemase (1∶4000, Abcam ab45434; lot434164) and mouse anti-GFAP (1∶800, Sigma G3893; lot080M4838) were applied and sections stored in a humid chamber overnight at 4°C. Sections were rinsed 3×5 minutes in PBS prior to incubation in a humid chamber with secondary antibodies (goat anti-rabbit Cy3-conjugated, 1∶600; goat anti-mouse Alexa488-conjugated, 1∶400; both from Invitrogen) for 1 hour in the dark. Sections were rinsed 3×5 minutes in PBS prior to coverslipping and mounting with Fluoromount G (Southern Biotech). Images were obtained using a Zeiss Imager.M2 epifluorescence microscope equipped with an Axiocam MRm camera and AxioVision software (rel. 4.8). Sections from both genotypes were always processed in parallel and imaged using the same exposure times. ZVI files were converted to TIFF format, deconvolved using the Iterative Deconvolution plugin by Bob Dougherty, and merged in Image J (version 1.44o, NIH). Figures were produced with Photoshop CS4 (v10.0.1, Adobe). Background levels were adjusted identically for both genotypes. Since there is no serine racemase expression in the spleen [42], [43] we used wild type spleen slices to confirm antibody specificity for the rabbit anti-serine racemase antibody (data not shown).

### High performance liquid chromatography analysis (HPLC)

Tissue samples were weighed after extraction and homocysteine as an internal standard was added. After that the samples were homogenized in 10 volumes of 5% trichloroacetic acid (TCA) and the homogenates were centrifuged at 18000×*g* at 4°C for 30 min. To remove TCA, the supernatants were washed three times with water-saturated diethyl ether. The resultant samples were used for HPLC analysis. Amino acid enantiomers were separated by HPLC using a carbon 18 reverse-phase column (250 mm) (Knauer, Advanced scientific instruments, Germany) with fluorimetric detection after derivatization with N-isobutyryl-L-cysteine and O-phthalaldehyde (OPA) as described in [44]. The N-isobutyryl-L-cysteine/OPA derivatives were immediately applied to the HPLC system (Knauer, Advanced scientific instruments, Germany). Mobile phase was 8% MeCN in 0.1 M sodium acetate buffer (pH 6.0). Amino acids were separated isocratically for 37 minutes and then the column was eluted with 50% H_2_O/50% MeCN for 5 minutes. After that, the column was reequillibrated for 15 minutes under the initial conditions. The flow-rate was 0.125 ml/min. Fluorescence detection of each amino acid derivative was carried out at 443 nm with excitation at 344 nm [44]. The absolute L-serine and D-serine levels referring to the internal standard were calculated and data were related to the wet weight of the initial tissue samples.

### Behavioral tests

Experiments were performed with 8–10 weeks old SrrTg from two independent founders (Srr12 and Srr2) and Wt littermates on a CD1 background. Animals received water and food *ad libitum* except during the operant conditioning period and the novelty-suppressed feeding paradigm. Mice were housed in groups of 3–5 animals and kept in a reversed light–dark cycle (with a dark period between 9:00 am and 6:00 pm). Experiments were conducted during the active phase of the animals in a dimly lit, low-noise environment. The experimenter was blind to the genotype. SrrTg mice were always compared to Wt littermates pooled from both transgenic lines. Care of the animals and performance of all experiments followed the guidelines of the German Animal Protection Law and were approved by the legal authorities (LANUV NRW, Permit number: 50.203.2-BN 34 2/06). The animals were left undisturbed for at least one week between testing when used in multiple models. The sequence of testing was as follows: Open-field test, Y-maze test, novelty-induced suppression of feeding and forced swim test. In the ultrasonic vocalisation test, bulbectomy-induced hypermotility paradigms and in the operant learning and Morris water maze models we used separate groups of animals that were tested in only one model, respectively. The home cages were brought into the test room at least 30 min before each experiment.

#### Forced swim test

The forced swim test is a commonly used procedure of preclinical screening of drugs for the antidepressant activity [45]. Animals were placed in a Plexiglas cylinder (10 cm inner diameter, 50 cm high) filled with 22–23°C water (20 cm height). The duration of the experiment was 6 min. The immobility time of the animals was evaluated between the 2nd and 6th minute for 4 min. A mouse was judged to be immobile when it remained floating in the water, making only movements necessary to keep its head above the water [45]–[47]. Means and SEM were calculated for each group.

#### Open-field test

Mice were placed into the centre of a dimly lit (20–30 lux) chamber of the open-field apparatus (44×44×30 cm). Movements of the animals were tracked by an automatic monitoring system (TSE Systems, Bad Homburg, Germany) for 30 min. Horizontal motor activity was evaluated by calculating the distance that the animals travelled in the arena. For each group, the mean value and SEM was calculated.

#### Novelty-suppressed feeding

For this test we used 20 Wt and 20 transgenic mice that were food deprived for 24 hr before testing. During testing, animals were placed individually close to a wall of an open-field apparatus lit by normal house lighting (light intensity 300–400 lux in the centre of the box). Six pellets (2.8–3.3 mg) of standard mouse chow were placed in the center of the open field. The latency to eat (nt sniffing or manipulating the pellets) was recorded by an observer who was unaware of the genotype [48].

#### Olfactory bulbectomy-induced hyperactivity

The bulbectomy was performed as described previously [49]. Mice were anesthetized with avertin (2.5%, 20 ml/kg). An incision was made in the skin overlying the skull, and a 2 mm hole was drilled into the skull through the frontal suture. Olfactory bulbs were removed by gentle aspiration. The same procedure was performed on sham-operated animals, except that the olfactory bulbs were not removed. Each animal was housed singly after surgery. Locomotor activity of the bulbectomized and sham-operated animals was studied 14 d after the surgery in a dimly lit, sound-attenuated room. The animals were placed in an open-field arena (45×45×22 cm) and the distance traveled was recorded for 5 min (Actimeter, TSE). After completion of the behavioral experiments, mice were decapitated, brains were removed, and the success of the OBX was evaluated visually.

#### Ultrasonic vocalization

Five to seven day-old pups were removed from their mother, placed individually into a glass jar, and put into a sound isolated box kept at room temperature (23±1°C). These pups emitted characteristic ultrasonic calls, which are known to be reduced by antidepressants [50]–[54]. The duration and number of these calls was registered automatically using the Ultravox system (Noldus, The Netherlands) for 5 min.

#### Y- maze

The Y-maze was composed of three equilaterally intersecting Plexiglas arms (58 cm long ×19 cm wide ×38 cm high). Mice were placed in the apparatus and arm visits were analyzed for 10 minutes using the Videomot 2 (TSE Systems, Bad Homburg, Germany) video observation system. Mean values and SEM were calculated for each group and expressed as percent spontaneous arm visits and the total number of arm visits.

#### Operant conditioning test

The day before and during the operant conditioning test the animals received only 80% of the food consumed under *ad libitum* conditions (Bilkei-Gorzo et al, 2005). Operant conditioning was developed by B.F. Skinner and is a type of learning in which organisms learn to voluntarily respond in a certain way depending on the consequences e.g. rewards [55]. Test cages (17×17×17 cm) were made from transparent plastic material and placed into a larger (50×40×70 cm) wooden box. Each cage contained a nose-poke sensor, a feeder and signal lamps for visual cues. The cages were connected to a computer-controlled central unit (TSE GmbH, Germany). Animals were placed individually into the cages, and number of nose-pokes into the sensor hole was registered for twenty minutes. Each nose-poke resulted in a delivery of a 50 mg food-pellet. The timeout period was 5 seconds and was signaled with a yellow lamp. The animals were tested daily until the variation between the responses on three consecutive days was less than 30%, but for a maximum of 24 days. Means and standard deviation of nose pokes were calculated daily for each group. Throughout the testing period the animals were single housed.

#### Morris Water maze test

In the Morris water maze we assessed hippocampal dependent spatial learning and memory abilities. One week before the experimental period, male mice (9–10 weeks old) were transferred to standard single-mouse cages, maintained at a 12:12-h inverted cycle (light on 07:00 pm–07:00 am), and tested during the dark period. The home cages were brought into the test room at least 30 min before each experiment. Water maze test was conducted with hemizygous transgenic animals, which were compared to their Wt littermates.

A circular pool (diameter 120 cm and height 30 cm) made of black PVC was used in a dimly illuminated room. The pool was filled to a depth of 15 cm with opacified water using non-toxic white paint (24°C). Four orthogonal starting positions were situated around the perimeter of the pool, dividing its surface into four quadrants. A platform in form of a transparent Plexiglas cylinder (15 cm tall and 8 cm diameter) covered with a white aluminum perforated plate (14 cm diameter) was placed in the center of one quadrant, approximately 1.5 cm under the water level and served as an escape platform. The pool was located in a room containing numerous extra-maze visual cues. A camera was fixed on the ceiling above the water maze.

#### Water maze procedure

Each mouse was tested for four consecutive sessions daily over 5–6 days. The hidden platform remained at a fixed spatial location for the entire acquisition period and each mouse was assigned a different escape sector. The mice were released, facing the wall of the maze at each trial session. During the first two days, animals were put into the same starting points (N) for each session. From day 3^rd^ to day 5^th^, animals were released into the four consecutive positions for each session (N, E, S, W).

A trial ended when the mouse reached the hidden platform and managed to remain there for 5 sec. If a successful escape did not take place within 70 sec, the mouse was guided to the platform and the trial was recorded as an escape failure with an arbitrary latency of 70 sec. The tested mouse was left for a 15 sec inter-trial interval in a dark dry container.

### D-serine supplementation

D-serine (Sigma), L-serine (Sigma) and glycine (Sigma) were dissolved in water and the pH was adjusted to 7.0. Wt mice consumed water, or water supplemented with 350 mg/L D-serine, L-serine, or glycine for 35 days. This resulted in a daily dose of 58 mg/kg D-serine per body weight (average weight: 30 g; average drinking volume: 5 ml/day). Preliminary studies using a two bottle choice test revealed no differences in preference for water alone or water supplemented with the amino acids used here. Although D-serine is more brain penetrant than L-serine and glycine, we used the latter amino acids to control for possible unspecific effects of the food supplementation. The liquid consumption was documented every second day before the liquid was replaced with freshly prepared solution. After the behavioral analyses all mice were sacrificed and the brains prepared, dissected and submitted to HPLC analysis.

### Statistical analysis

Behavioral data analysis for males and females are provided separately even though there was no effect of sex nor a sex x genotype interaction. Data were analyzed, where appropriate, by paired or unpaired t-test, one-way, two-way/three-way or repeated measures analysis of variance (ANOVA) followed by either Dunnett's- or Bonferroni *post hoc* tests. If the data were not normally distributed, we applied non-parametric tests for analysis (Kruskal–Wallis test). Analysis was done by using the GraphPad Prism version 4.00 for MAC or by Statistica version 7.1. Unless otherwise indicated, data are expressed as mean ±SEM, and statistical significance was considered when p<0.05.

## Results

### Generation of GFAP-Srr transgenic mouse strains

We established two transgenic mouse lines, Srr2 and Srr12, harboring the serine racemase ORF under the control of the GFAP-promoter (Fig. 1A). Quantitative real-time PCR with mRNA from whole brain extracts showed that both lines expressed the transgene, with higher levels in the Srr12 strain (Fig. 1B, p<0.001). *Western* blot analysis of whole brain protein lysates from Srr12 transgenic and Wt animals also revealed a higher serine racemase protein content (Fig. 1C) and concomitantly increased absolute D-serine levels in different brain regions, except for the forebrain of Srr12 mice (Fig. 1D, two-way ANOVA: hippocampus: genotype F(1,16) = 8.89, p = 0.0088; cortex: genotype F(1,16) = 6.48, p = 0.0216; amino acid F(1,16) = 14.10, p = 0.0017, interaction: F(1,16) = 6.08, 0.0254; forebrain: genotype F(1,13) = 1.41, p = 0.2556; amino acid F(1,13) = 11.19, p = 0.0053, interaction: F(1,13) = 6.22, p = 0.0269).

**Figure 2:**
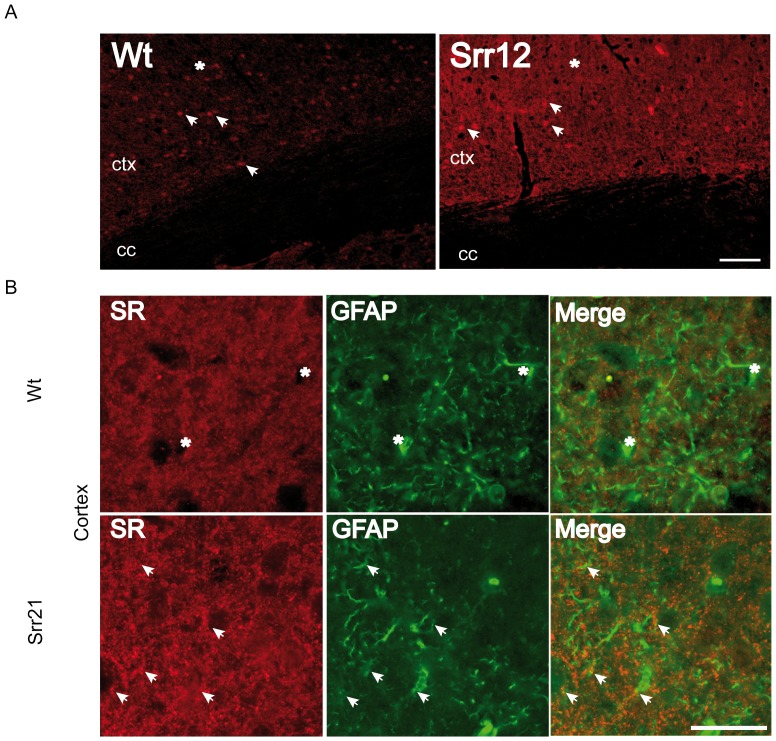
Specific serine racemase immunofluorescence in Srr12 mice. (A) Immunoreactivity for serine racemase is higher in transgenic animals than in Wt controls. Arrows - indicate serine racemase positive cell soma. * - indicates immunoreactivity of the neuropil which is much higher in Srr12 transgenic mice than Wt controls, possibily due to higher expression in astrocytic processes. (B) Serine racemase expression is specifically increased in astrocytes as labeled by the astrocyte marker glial fibrillary acidic protein (GFAP). Arrowheads - indicate GFAP^+^ processes bearing SR^+^ puncta, although both Wt and Srr12 astrocytes express serine racemase, immunoreactivity and colocalization is more pronounced in Srr12 mice. Asterisks indicate GFAP positive cells which seemed to be devoid of serine racemase expression in WT mice. All images were acquired using the same exposure times. Scale bar - 100 µm top panels and 50 µm all other panels. ctx - cortex, cc - corpus callosum.

Immunofluorescence analysis of the Srr12 mouse line revealed stronger immunoreactivity in the cortex of Srr12 mice, especially within the neuropil (Fig. 2A, main panels). Although GFAP^+^ cells express serine racemase in wildtype animals, the expression is much higher in Srr12 mice as demonstrated for the cortex (Fig. 2A/B). This is quite noticeable in the Srr12 cortex where serine racemase immunoreactivity localizes to GFAP^+^ astrocytes (arrows) whereas Wt astrocytes seemed to be at least partially devoid of serine racemase expression (asterisks).

We observed no gross abnormalities, reproductive deficits or behavioral abnormalities in transgenic animals by routine handling and therefore conducted a series of behavioral tests to determine if the elevated D-serine levels affect distinct mouse behaviors. Although we examined both lines in parallel in many behavioral tests, we conducted some experiments only with Srr12 mice, because they generally showed a slightly more pronounced phenotype.

### Mood-related behaviors in SrrTg mice

**Figure 3:**
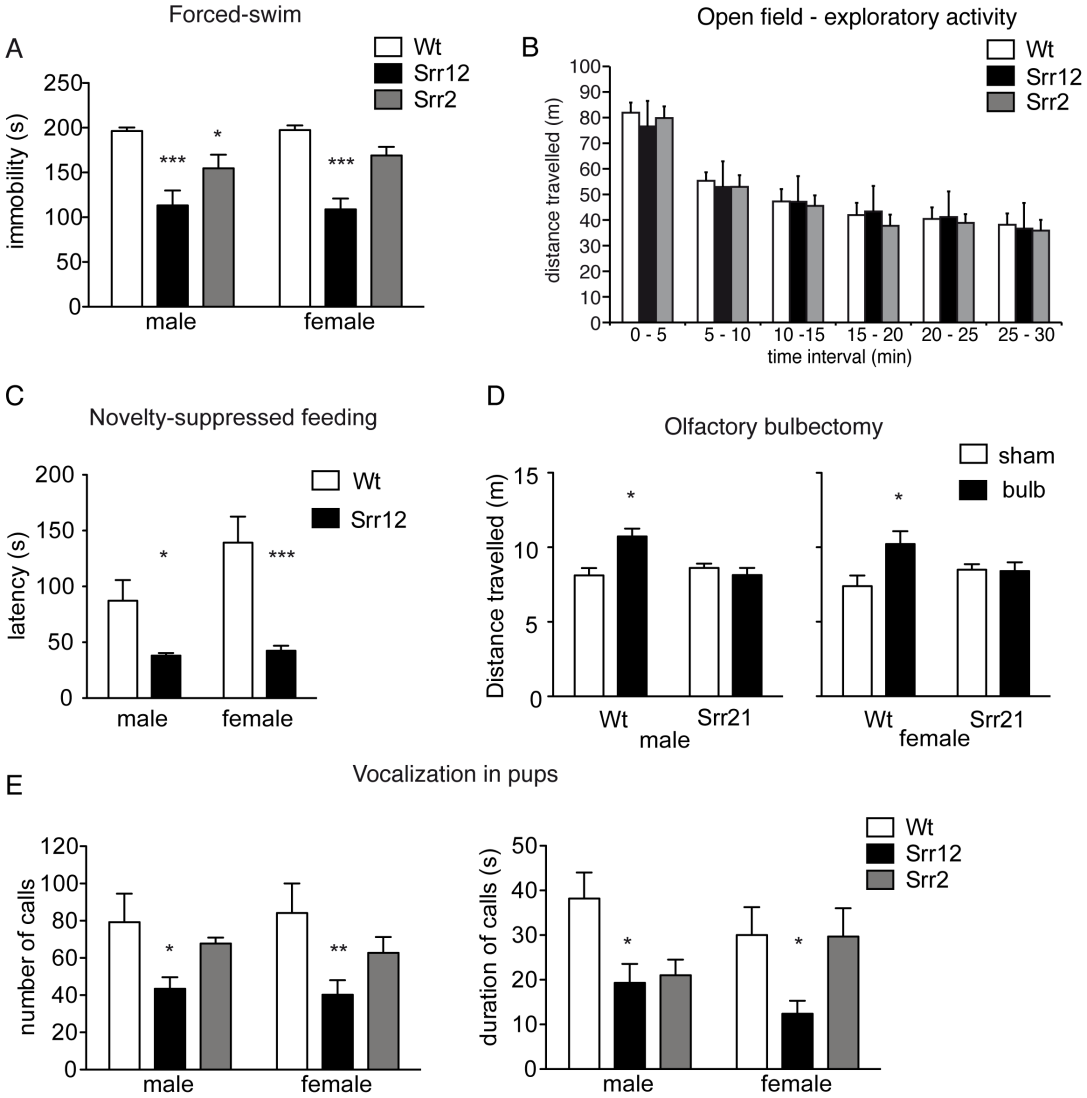
Depression-related behavior in SrrTg mice. (A) Forced-swim test. Immobility time, defined as a lack of activity aside from small movements needed to keep the body floating, was measured throughout the last 4 min of the session. (Wt: n = 21 (10 males, 11 females); Srr2: n = 17 (8 males, 9 females); Srr12: n = 24 (9 males, 15 females)). (B) Locomotor activity was recorded over a 30 min time period and expressed as distance traveled. (n = 15 per group). (C) Novelty suppressed feeding test. Srr12 mice displayed a significantly reduced latency to approach and eat food compared to Wt mice (Wt: n = 26 (14 males, 12 females); Srr12: n = 28 (15 males, 13 females)). (D) Horizontal (distance traveled) activity 2 weeks after surgery of sham-operated and bulbectomized SrrTg mice and their sham operated littermates in the open-field apparatus (Wt: female 16 (sham 10, bulb 6), male 18 (sham 14, bulb 4); Srr12: female 15 (sham 8, bulb 7), male 13 (sham 5, bulb 7). (E) Ultrasonic vocalization. Measurements of social isolation-induced ultrasonic vocalizations (USV) in SrrTg and Wt pups revealed a significant increase in Srr12 mice in number (left) and duration (right) of calls (Wt: n = 22 (12 males, 10 females); Srr2: n = 23 (9 males, 13 females); Srr12: n = 35 (20 males, 15 females)). All values are expressed as mean ±SEM. *p<0.05 and ***p<0.001.

In the Porsolt forced swim test, we observed a significantly reduced immobility time in both transgenic lines compared to Wt animals (Fig. 3A, Kruskal–Wallis test = 28.29, p<0.0001). We did not find any sex (two-way ANOVA F(1,56) = 0.15, p = 0.7) or sex genotype interaction (two-way ANOVA F(2,56) = 0.32, p = 0.72,). Furthermore, we assessed exploratory behavior of Srr12 and Srr2 mice in the open field test. Both transgenic lines exhibited a similar exploratory activity and habituation during the 30 minutes observation time as Wt mice (three-way ANOVA genotype effect, F(2,80) = 0,312, p>0.77; time effect F(5,395) = 52,435, p>0.01; Fig. 3B). In the novelty-suppressed feeding (NSF) test, Srr12 animals showed a highly significant reduction in the latency to approach the food compared to Wt mice (Fig. 3C, one-way ANOVA F(1,28) = 16.89, p<0.01), whereas the general food intake in both transgenic mouse strains was not changed (data not shown) Again, we did not observe any sex (two-way ANOVA F(1,50) = 3.86, p = 0.055) or sex genotype interaction (two-way ANOVA F(1,50) = 2.78, p = 0.10). We also analyzed the effects of a bilateral olfactory bulbectomy (OBX), an animal model for depression, in Wt and Srr12 transgenic mice. We found a significant genotype x surgery interaction for both sexes (two-way ANOVA male F(1,26) = 6.39. p<0.05; two-way ANOVA female F(1,26) = 12.89 p<0.05). OBX produced a characteristic hyperactivity, indicated by an increased distance traveled during the first 6 minutes in the open field (Fig. 3D) in Wt mice, but not in Srr12 transgenic mice. Finally, we analyzed the SrrTg mice in the ultrasonic vocalization test (USV). Transgenic pups of the Srr12 line displayed a significant reduction in the number (Fig. 3E left, one-way ANOVA F(2,44) = 3.31, p<0.05) and in the duration of calls (Fig. 3E right, p<0.01, Kruskal–Wallis test). We did not notice any sex (number of calls: two-way ANOVA F(1,73) = 0.03, p = 0.90; duration of calls: two-way ANOVA F(1,73) = 0.25, p = 0.62) or sex genotype interaction (number of calls: two-way ANOVA F(2,73) = 0.14, p = 0.87; duration of calls: two-way ANOVA F(2,73) = 1.53, p = 0.22). The behavior of Srr2 pups in this paradigm was not different from Wt pups.

### Cognition and memory in SrrTg mice

**Figure 4:**
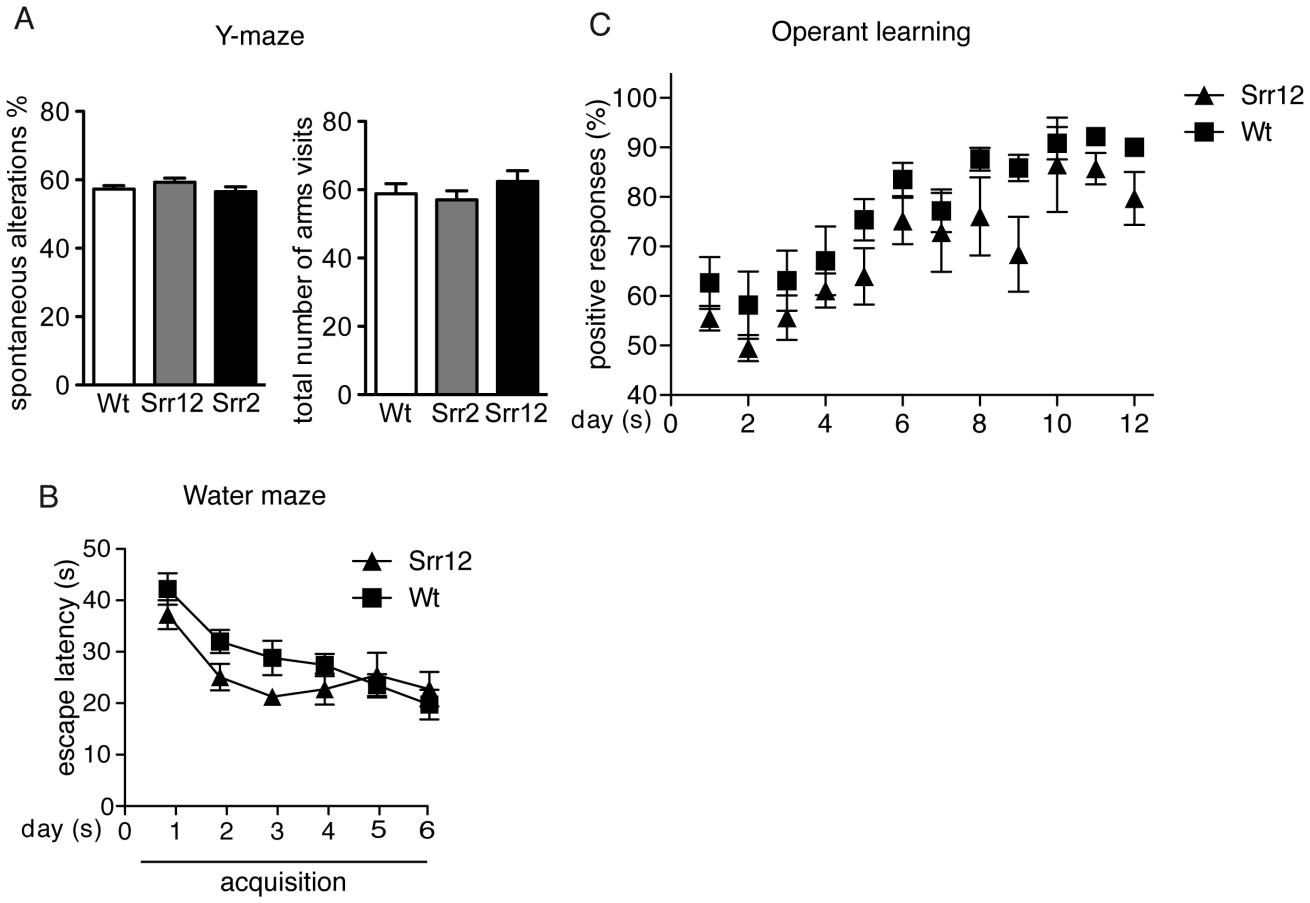
Cognition in SrrTg mice. (A) In the Y-maze task, SrrTg mice displayed no significant differences in the percentage of spontaneous alternations (left) and total number of arm visits (right) compared to Wt mice (Wt: n = 24; Srr2: n = 19; Srr12: n = 27). (B) Morris water maze test. Average water maze escape latencies across 5 training days of Wt and Srr12 mice (n 9–10 male mice per group). (C) Operant conditioning test. The learning performance of SrrTg mice did not differ compared to Wt mice (Wt: n = 16; Srr12: n = 16). All values are expressed as mean ±SEM indicated animals.

The Y-maze test assesses spatial working memory as percentage of spontaneous alternations, calculated as the number of alternations (entering into three different arms consecutively) divided by the number of possible alternations (total arm visits minus 2) and multiplied with 100. Neither the spontaneous alternation (Fig. 4A, left) or the total number of arm visits (Fig. 4A, right) differed significantly between Srr12, Srr2 and Wt littermates (Fig. 4A left, one-way ANOVA, same: F(2,69) = 1.204, p>0.05; Fig. 4A right: F(2,69) = 0.8257, p>0.05).

The Morris water maze is widely used to study spatial memory and learning [56]. Repeated measures ANOVA for the latency to find the platform during acquisition (day 1–5) revealed that overall both Wt and Srr12 mice learned to find the hidden platform, which was indicated by a significant time effect for both strains (F(5,85) = 11.01, p<0.0001, Fig. 4B). Srr12 mice did not perform differently from their Wt littermates (F(1,17) = 2,87, p = 0.11) and there was no genotype x time interaction (F(5,85) = 1.35, p = 0.25). The swim speed was neither affected by genotype (F(1,17) = 0.00, p = 0.9) nor by time (F(4,68) = 0.75, p = 0.8). Additionally, no significant genotype x time interaction was found (F(4,68) = 2.04, p = 0.10).

We also studied cognitive functions in an operant learning paradigm (Fig. 4C). Repeated measures ANOVA revealed that Srr12 mutant mice did not show altered operant learning abilities compared to Wt mice. There was a significant effect of time (F(11,308) = 29.22, p<0.001), but no genotype effect (F(1,28) = 2.162, p>0.05) and no time x genotype interaction (F(11,308) = 1.177, p>0.05).

### D-serine supplementation

**Figure 5:**
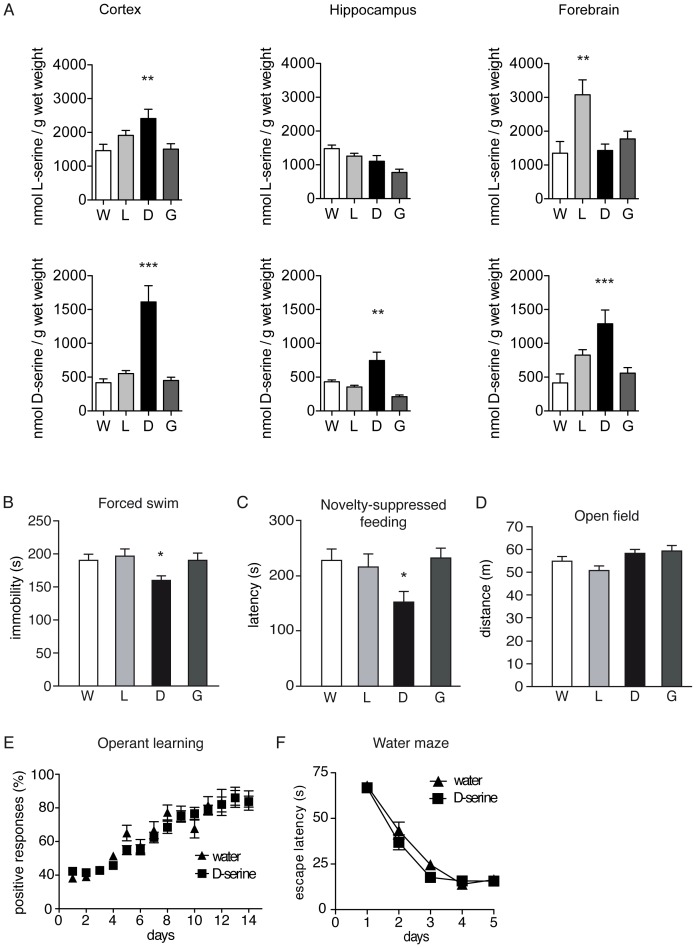
Brain D-serine levels and behavior of Wt mice after D-serine administration. (A) Quantitative analysis of brain D-serine and L-serine levels per wet weight in 4–8 dietary-treated Wt mice by HPLC. D-serine levels of cortex, hippocampus and forebrain were measured. Mice were treated with D-serine (D), L-serine (L), glycine (G) and water (W). (B) Forced-swim test. Immobility time, defined as a lack of activity aside from small movements needed to keep the body floating, was measured throughout the last 4 min of the session. D-serine treated mice spent less time immobile compared to controls. (C) Novelty suppressed feeding test. The mice that were treated with D-serine displayed a significantly reduced latency to approach the food and to start eating. (D) Open field test. Distance traveled in the open field arena of Wt mice. Behavior in this paradigm was not changed after amino acid treatment. (E) Operant conditioning test. The Hill equation indicates learning improvement during the training days. Data of all groups resulted in similar learning curves. (F) Water maze test. Escape latencies across 5 training days of D-serine treated and control mice were recorded and did not differ between the groups. *p<0.05 and **p<0.01. Each error bar represents the mean ±SEM of 10 animals.

We investigated the consequences of a dietary D-serine drinking supplementation in Wt mice. Accordingly, we added 350 mg/kg D-serine to the drinking water for a 5-week period. Control mice either received water, L-serine or glycine. One-way ANOVA revealed that mice receiving D-serine had considerably higher brain D-serine levels compared to the control groups in all brain regions studied (one-way ANOVA, Fig 5A, cortex F(3,22) = 18.17, p<0.0001; hippocampus F(3,22) = 14.15, p<0.0001; forebrain F(3,22) = 8.872, p<0.0004). Additionally, D-serine treatment led to significantly increased L-serine levels in the cortex and L-serine treatment significantly increased the L-serine levels in the forebrain (Fig 5A). Behavioral testing showed that mice receiving D-serine had a significantly reduced immobility time in the Porsolt forced swim test when compared to the water control group (one-way ANOVA, F(3,46) = 3,747, p<0.05, Fig. 5B). L-serine or glycine administration had no effect. The latency to approach food in the novelty-suppressed feeding test was also significantly decreased in mice receiving D-serine (one-way ANOVA, F(4,59) = 2,812, p<0.05, Fig. 5C). Again, there was no effect of L-serine or glycine supplementation. The open field test revealed no differences in the distance travelled between the different groups indicated that D-serine does not influence open field activity (one-way ANOVA Fig. 5C) The operant learning test revealed that a chronic D-serine treatment had no influence on learning behavior in this paradigm. Both groups, D-serine treated and controls, performed equally (two-way repeated ANOVA, time effect: F(13,494) = 54.86, p<0.0001; treatment effect: F(1,38) = 0.19, p<0.66; Fig. 5E). In the water maze test, D-serine treated mice behaved similarly compared to the control group (two-way ANOVA repeated measurers, time effect: F(4,72) = 158.83, p< <0.0001; treatment effect: F(1,18) = 1.45, p<0.24; Fig. 5F. Thus, the dietary and genetic elevation of D-serine levels produced similar behavioral effects in depression- related and cognitive paradigms as those observed in SrrTg animals.

## Discussion

In this study we investigated the consequences of genetically or pharmacologically increased brain D-serine levels on animal behavior. Both the astrocytic expression of serine racemase in transgenic mice or the supplementation of the drinking water with D-serine resulted in significantly elevated brain D-serine levels in the cortex and the hippocampus. This affected emotional behaviors but not the cognitive performance of the mice. SrrTg mice displayed increased serine racemase protein expression shown by *Western* blot and immunofluorescence analyses. This increased serine racemase expression co-localized with GFAP expression, which is an astrocytic marker in the brain [42], [57]–[60]. This co-localization indicated that SrrTg mice indeed have augmented astrocytic serine racemase expression.

### Chronic D-serine elevation results in antidepressive behavior

The Porsolt forced swim test evaluates behavioral despair in mice. SrrTg mice and animals chronically treated with D-serine showed a reduced immobility time in this paradigm, thus behaving like animals treated with antidepressant drugs. This behavior was not due to a generally elevated activity in these mice, because they showed no changes in locomotor activity in the open field test. Hence, it is more likely that the elevated D-serine levels altered affective or depression-related behaviors in these animals. These findings are in line with emerging evidence that glutamatergic signaling may be involved in the pathogenesis of major depressive disorders [61], although the mechanisms are still not completely understood. Accordingly, a single application of the NMDA-receptor selective antagonist ketamine, a widely used anesthetic drug, can produce a long-lasting antidepressant effect. This effect seems to be mediated through an up-regulation of mammalian target of rapamycin (mTor) signaling [62], [63]. There is also evidence that ketamine enhances glutamatergic signaling in the prefrontal cortex, at least at low doses [64], [65],[66]. D-cycloserine (DCS), a partial agonist at the strychnine-insensitive glycine site of the NMDA receptor [37], also showed antidepressant effects in animal models [67] and in humans [68], which is in line with our observation of antidepressant-like behavior in the transgenic mice with elevated brain D-serine levels. The influence of low D-serine levels on depression-like behavior has not been tested so far as serine racemase-deficient mice have not been examined in behavioral paradigms of depression. They display an anxiety-related behavioral phenotype with cognitive deficits [39].

We also investigated the consequences of an olfactory bulbectomy (OBX) in SrrTg mice. Removal of the *bulbus olfactorius* in rodents is considered as an animal model of depression with high predictive validity, because it triggers physiological and behavioral changes that are sensitive to antidepressant treatment, but not to anxiolytic drugs [69]. Behavioral deficits in bulbectomized mice are accompanied by NMDA receptor-mediated dysfunctions in the hippocampus and amygdala [70]. It has been suggested that these dysfunctions are due to a disconnection of projections from the olfactory bulb to the olfactory cortex, including the olfactory tubercle, amygdala, and entorhinal cortex after bulbectomy [71]. While Wt mice displayed an elevated locomotor activity in the open field after OBX, we could not observe such behavioral effects in SrrTg mice. Together these findings strongly suggest that chronically elevated D-serine levels protect against the behavioral changes in the OBX mouse model of depression.

The USV test evaluates distress-like ultrasonic calls that pups emit after separation from their mothers. This behavior is considered as an expression of affect and is used for the preclinical evaluation of anxiolytic drugs [72]–[74]. SrrTg pups showed a significantly lower number and shortened duration of USV, again indicating an effect of elevated D-serine levels on affective responses. Glutamatergic signaling has been implicated in this behavior, as USVs were enhanced after administration of low to moderate doses of the NMDA receptor antagonists memantine or neramexane, while higher doses reduced USVs [75]. The high-affinity NMDA receptor antagonist dizocilpine reduced USVs, without showing biphasic dose-response effects [75]. Additionally, 1-aminocyclopropanecarboxylic acid (ACPC), a partial agonist on the NMDA receptor, reduces USV in rats, whereas glycine does not alter USV [76]. Despite these results the mechanism by which D-serine alters USV in SrrTg mice remains unclear.

We also evaluated SrrTg mice and D-serine-treated mice in the NSF test, a conflict paradigm with a food reward being the positive reinforcer and fear in a novel environment the negative reinforcer. This test is sensitive to chronic antidepressant treatment, as well as to anxiolytic drugs [77]. Again, SrrTg and D-serine-treated mice showed altered emotionality, as they approached the food significantly faster than control animals.

Interestingly, we observed a more pronounced behavioral phenotype in serine racemase overexpressing mice, when compared to animals that received D-serine orally, although the latter animals exhibited higher brain D-serine levels, albeit not measured at the same time. This is probably due to the fact that astrocytic D-serine is more efficient in enhancing NMDA receptor signaling than D-serine provided via drinking water. Astrocytic D-serine is released by a vesicle-dependent mechanism that is closely related to glutamate release and calcium influx and thus regulated by neuronal activity [78].

### Mild chronic D-serine elevation and cognition

350 mg/L D-serine added to the drinking water increased D-serine levels in all brain regions studied. That D-serine treatment at least acutely increases D-serine content in mice and rats has already been demonstrated [79], [80]. Moreover, D-serine treatment also increases L-serine levels in the cortex indicating that D-serine is possibly converted to L-serine by the serine racemase in this brain region [3], [24]. Studies investigating the effects of D-serine administration on cognitive performances showed different results. Thus, an acutely applied dose of 1000 mg/kg D-serine to rats led to cognitive enhancement in the Morris water maze test, while a dose of 100 mg/kg showed no effect [81]. A single administration of 800 mg/kg D-serine also enhanced social memory in rats [82]. These studies suggested that only higher doses of D-serine enhance the cognitive performance of rats. However, functional magnetic resonance imaging demonstrated that administration of 50 mg/kg of D-serine is enough to enhance hippocampal activity in rats [83]. Therefore, Bado et al., examined a 50 mg/kg dose of D-serine applied prior to cognitive test situations and showed that this low dose effectively enhanced cognitive performances in several tasks [79]. In this study, we investigated the effects of a chronic low dose (58 mg/kg/d) D-serine treatment on the cognitive performance in mice. This treatment had no effect on the animals' performances in the operant conditioning paradigm or the Morris water maze test. Likewise, the performance of SrrTg animals was also not significantly different from Wt littermates in these tests.

It has recently been demonstrated that the GFAP promoter is also present in slowly cycling, stationary cells of the brain that develop into intermediate progenitors [84]. We therefore cannot exclude that the serine racemase is also expressed in these neuronal progenitors of SrrTg mice. However, D-serine treatment also produced the same behavioral changes observed in SrrTg mice and, therefore, it is unlikely that the observed behavioral changes are caused by developmental effects.

Our present data show that a chronic elevation of D-serine levels, either by overexpression of serine racemase or by drinking supplementation, improves the emotional behavior of mice. It should be noted that the total brain D-serine levels were only significantly changed under our experimental conditions, with up to 3-fold elevation in the SrrTg mice and after food supplementation of Wt mice. The D-serine increase was most pronounced in the cortex area of both models. We suggest D-serine as useful for the pharmacotherapy of mood disorders.

## References

[1] Li F, Tsien JZ (2009) Memory and the NMDA receptors. N Engl J Med 361: 302–303. 19605837 10.1056/NEJMcibr0902052 PMC3703758

[2] Kemp JA, Leeson PD (1993) The glycine site of the NMDA receptor–five years on. Trends Pharmacol Sci 14: 20–25. 8382885 10.1016/0165-6147(93)90108-v

[3] Wolosker H, Blackshaw S, Snyder SH (1999) Serine racemase: a glial enzyme synthesizing D-serine to regulate glutamate-N-methyl-D-aspartate neurotransmission. Proc Natl Acad Sci U S A 96: 13409–13414. 10557334 10.1073/pnas.96.23.13409 PMC23961

[4] Baranano DE, Ferris CD, Snyder SH (2001) Atypical neural messengers. Trends Neurosci 24: 99–106. 11164940 10.1016/s0166-2236(00)01716-1

[5] Mothet JP, Parent AT, Wolosker H, Brady RO Jr, Linden DJ, et al (2000) D-serine is an endogenous ligand for the glycine site of the N-methyl-D-aspartate receptor. Proc Natl Acad Sci U S A 97: 4926–4931. 10781100 10.1073/pnas.97.9.4926 PMC18334

[6] Schell MJ, Brady RO Jr, Molliver ME, Snyder SH (1997) D-serine as a neuromodulator: regional and developmental localizations in rat brain glia resemble NMDA receptors. J Neurosci 17: 1604–1615. 9030620 10.1523/JNEUROSCI.17-05-01604.1997 PMC6573391

[7] Schell MJ, Molliver ME, Snyder SH (1995) D-serine, an endogenous synaptic modulator: localization to astrocytes and glutamate-stimulated release. Proc Natl Acad Sci U S A 92: 3948–3952. 7732010 10.1073/pnas.92.9.3948 PMC42079

[8] Kim PM, Aizawa H, Kim PS, Huang AS, Wickramasinghe SR, et al (2005) Serine racemase: activation by glutamate neurotransmission via glutamate receptor interacting protein and mediation of neuronal migration. Proc Natl Acad Sci U S A 102: 2105–2110. 15684087 10.1073/pnas.0409723102 PMC548584

[9] Berger AJ, Dieudonne S, Ascher P (1998) Glycine uptake governs glycine site occupancy at NMDA receptors of excitatory synapses. J Neurophysiol 80: 3336–3340. 9862928 10.1152/jn.1998.80.6.3336

[10] Martina M, Krasteniakov NV, Bergeron R (2003) D-Serine differently modulates NMDA receptor function in rat CA1 hippocampal pyramidal cells and interneurons. J Physiol 548: 411–423. 12611916 10.1113/jphysiol.2002.037127 PMC2342854

[11] Lim R, Hoang P, Berger AJ (2004) Blockade of glycine transporter-1 (GLYT-1) potentiates NMDA receptor-mediated synaptic transmission in hypoglossal motorneurons. J Neurophysiol 92: 2530–2537. 15175365 10.1152/jn.01123.2003

[12] Kinney GG, Sur C, Burno M, Mallorga PJ, Williams JB, et al (2003) The glycine transporter type 1 inhibitor N-[3-(4′-fluorophenyl)-3-(4′-phenylphenoxy)propyl]sarcosine potentiates NMDA receptor-mediated responses in vivo and produces an antipsychotic profile in rodent behavior. J Neurosci 23: 7586–7591. 12930797 10.1523/JNEUROSCI.23-20-07586.2003 PMC6740762

[13] Chen L, Muhlhauser M, Yang CR (2003) Glycine tranporter-1 blockade potentiates NMDA-mediated responses in rat prefrontal cortical neurons in vitro and in vivo. J Neurophysiol 89: 691–703. 12574447 10.1152/jn.00680.2002

[14] Bergeron R, Meyer TM, Coyle JT, Greene RW (1998) Modulation of N-methyl-D-aspartate receptor function by glycine transport. Proc Natl Acad Sci U S A 95: 15730–15734. 9861038 10.1073/pnas.95.26.15730 PMC28112

[15] Hashimoto A, Nishikawa T, Hayashi T, Fujii N, Harada K, et al (1992) The presence of free D-serine in rat brain. FEBS Lett 296: 33–36. 1730289 10.1016/0014-5793(92)80397-y

[16] Hashimoto A, Nishikawa T, Oka T, Takahashi K (1993) Endogenous D-serine in rat brain: N-methyl-D-aspartate receptor-related distribution and aging. J Neurochem 60: 783–786. 8419554 10.1111/j.1471-4159.1993.tb03219.x

[17] Matsui T, Sekiguchi M, Hashimoto A, Tomita U, Nishikawa T, et al (1995) Functional comparison of D-serine and glycine in rodents: the effect on cloned NMDA receptors and the extracellular concentration. J Neurochem 65: 454–458. 7790891 10.1046/j.1471-4159.1995.65010454.x

[18] Priestley T, Laughton P, Myers J, Le Bourdelles B, Kerby J, et al (1995) Pharmacological properties of recombinant human N-methyl-D-aspartate receptors comprising NR1a/NR2A and NR1a/NR2B subunit assemblies expressed in permanently transfected mouse fibroblast cells. Mol Pharmacol 48: 841–848. 7476914

[19] Thomson AM, Walker VE, Flynn DM (1989) Glycine enhances NMDA-receptor mediated synaptic potentials in neocortical slices. Nature 338: 422–424. 2538754 10.1038/338422a0

[20] Yang Y, Ge W, Chen Y, Zhang Z, Shen W, et al (2003) Contribution of astrocytes to hippocampal long-term potentiation through release of D-serine. Proc Natl Acad Sci U S A 100: 15194–15199. 14638938 10.1073/pnas.2431073100 PMC299953

[21] Stevens ER, Esguerra M, Kim PM, Newman EA, Snyder SH, et al (2003) D-serine and serine racemase are present in the vertebrate retina and contribute to the physiological activation of NMDA receptors. Proc Natl Acad Sci U S A 100: 6789–6794. 12750462 10.1073/pnas.1237052100 PMC164525

[22] Baptista V, Varanda WA (2005) Glycine binding site of the synaptic NMDA receptor in subpostremal NTS neurons. J Neurophysiol 94: 147–152. 15744010 10.1152/jn.00927.2004

[23] Papouin T, Ladepeche L, Ruel J, Sacchi S, Labasque M, et al (2012) Synaptic and extrasynaptic NMDA receptors are gated by different endogenous coagonists. Cell 150: 633–646. 22863013 10.1016/j.cell.2012.06.029

[24] Wolosker H, Sheth KN, Takahashi M, Mothet JP, Brady RO Jr, et al (1999) Purification of serine racemase: biosynthesis of the neuromodulator D-serine. Proc Natl Acad Sci U S A 96: 721–725. 9892700 10.1073/pnas.96.2.721 PMC15203

[25] De Miranda J, Santoro A, Engelender S, Wolosker H (2000) Human serine racemase: moleular cloning, genomic organization and functional analysis. Gene 256: 183–188. 11054547 10.1016/s0378-1119(00)00356-5

[26] De Miranda J, Panizzutti R, Foltyn VN, Wolosker H (2002) Cofactors of serine racemase that physiologically stimulate the synthesis of the N-methyl-D-aspartate (NMDA) receptor coagonist D-serine. Proc Natl Acad Sci U S A 99: 14542–14547. 12393813 10.1073/pnas.222421299 PMC137919

[27] Benneyworth MA, Li Y, Basu AC, Bolshakov VY, Coyle JT (2012) Cell selective conditional null mutations of serine racemase demonstrate a predominate localization in cortical glutamatergic neurons. Cell Mol Neurobiol 32: 613–624. 22362148 10.1007/s10571-012-9808-4 PMC4817353

[28] Henneberger C, Papouin T, Oliet SH, Rusakov DA (2010) Long-term potentiation depends on release of D-serine from astrocytes. Nature 463: 232–236. 20075918 10.1038/nature08673 PMC2807667

[29] Coyle JT (2006) Glutamate and schizophrenia: beyond the dopamine hypothesis. Cell Mol Neurobiol 26: 365–384. 16773445 10.1007/s10571-006-9062-8 PMC11881825

[30] Coyle JT, Tsai G, Goff D (2003) Converging evidence of NMDA receptor hypofunction in the pathophysiology of schizophrenia. Ann N Y Acad Sci 1003: 318–327. 14684455 10.1196/annals.1300.020

[31] Hashimoto K, Sawa A, Iyo M (2007) Increased levels of glutamate in brains from patients with mood disorders. Biol Psychiatry 62: 1310–1316. 17574216 10.1016/j.biopsych.2007.03.017

[32] Bendikov I, Nadri C, Amar S, Panizzutti R, De Miranda J, et al (2007) A CSF and postmortem brain study of D-serine metabolic parameters in schizophrenia. Schizophr Res 90: 41–51. 17156977 10.1016/j.schres.2006.10.010

[33] Duncan EJ, Szilagyi S, Schwartz MP, Bugarski-Kirola D, Kunzova A, et al (2004) Effects of D-cycloserine on negative symptoms in schizophrenia. Schizophr Res 71: 239–248. 15474895 10.1016/j.schres.2004.03.013

[34] Heresco-Levy U, Kremer I, Javitt DC, Goichman R, Reshef A, et al (2002) Pilot-controlled trial of D-cycloserine for the treatment of post-traumatic stress disorder. Int J Neuropsychopharmacol 5: 301–307. 12466030 10.1017/S1461145702003061

[35] Laake K, Oeksengaard AR (2002) D-cycloserine for Alzheimer's disease. Cochrane Database Syst Rev CD003153. 12076471 10.1002/14651858.CD003153 PMC6718229

[36] Kantrowitz JT, Malhotra AK, Cornblatt B, Silipo G, Balla A, et al (2010) High dose D-serine in the treatment of schizophrenia. Schizophr Res 121: 125–130. 20541910 10.1016/j.schres.2010.05.012 PMC3111070

[37] Lopes T, Neubauer P, Boje KM (1997) Chronic administration of NMDA glycine partial agonists induces tolerance in the Porsolt swim test. Pharmacol Biochem Behav 58: 1059–1064. 9408214 10.1016/s0091-3057(97)00302-x

[38] Onur OA, Schlaepfer TE, Kukolja J, Bauer A, Jeung H, et al (2010) The N-methyl-D-aspartate receptor co-agonist D-cycloserine facilitates declarative learning and hippocampal activity in humans. Biol Psychiatry 67: 1205–1211. 20303474 10.1016/j.biopsych.2010.01.022

[39] Basu AC, Tsai GE, Ma CL, Ehmsen JT, Mustafa AK, et al (2009) Targeted disruption of serine racemase affects glutamatergic neurotransmission and behavior. Mol Psychiatry 14: 719–727. 19065142 10.1038/mp.2008.130 PMC2786989

[40] Malkesman O, Austin DR, Tragon T, Wang G, Rompala G, et al (2011) Acute d-serine treatment produces antidepressant-like effects in rodents. Int J Neuropsychopharmacol 1–14. 10.1017/S1461145711001386 PMC3278496 21906419

[41] Nolte C, Matyash M, Pivneva T, Schipke CG, Ohlemeyer C, et al (2001) GFAP promoter-controlled EGFP-expressing transgenic mice: a tool to visualize astrocytes and astrogliosis in living brain tissue. Glia 33: 72–86. 11169793

[42] Wang LZ, Zhu XZ (2003) Spatiotemporal relationships among D-serine, serine racemase, and D-amino acid oxidase during mouse postnatal development. Acta Pharmacol Sin 24: 965–974. 14531937

[43] Horio M, Kohno M, Fujita Y, Ishima T, Inoue R, et al (2011) Levels of D-serine in the brain and peripheral organs of serine racemase (Srr) knock-out mice. Neurochem Int 59: 853–859. 21906644 10.1016/j.neuint.2011.08.017

[44] Grant SL, Shulman Y, Tibbo P, Hampson DR, Baker GB (2006) Determination of d-serine and related neuroactive amino acids in human plasma by high-performance liquid chromatography with fluorimetric detection. J Chromatogr B Analyt Technol Biomed Life Sci 844: 278–282. 10.1016/j.jchromb.2006.07.022 16890503

[45] Bilkei-Gorzo A, Michel K, Noble F, Roques BP, Zimmer A (2007) Preproenkephalin knockout mice show no depression-related phenotype. Neuropsychopharmacology 32: 2330–2337. 17375141 10.1038/sj.npp.1301370

[46] Porsolt RD, Le Pichon M, Jalfre M (1977) Depression: a new animal model sensitive to antidepressant treatments. Nature 266: 730–732. 559941 10.1038/266730a0

[47] Porsolt RD, Bertin A, Jalfre M (1977) Behavioral despair in mice: a primary screening test for antidepressants. Arch Int Pharmacodyn Ther 229: 327–336. 596982

[48] Rochford J, Beaulieu S, Rousse I, Glowa JR, Barden N (1997) Behavioral reactivity to aversive stimuli in a transgenic mouse model of impaired glucocorticoid (type II) receptor function: effects of diazepam and FG-7142. Psychopharmacology (Berl) 132: 145–152. 9266611 10.1007/s002130050330

[49] Bilkei-Gorzo A, Racz I, Michel K, Zimmer A (2002) Diminished anxiety- and depression-related behaviors in mice with selective deletion of the Tac1 gene. J Neurosci 22: 10046–10052. 12427862 10.1523/JNEUROSCI.22-22-10046.2002 PMC6757849

[50] Noirot E (1972) Ultrasounds and maternal behavior in small rodents. Dev Psychobiol 5: 371–387. 4609822 10.1002/dev.420050410

[51] Brunelli SA, Shair HN, Hofer MA (1994) Hypothermic vocalizations of rat pups (Rattus norvegicus) elicit and direct maternal search behavior. J Comp Psychol 108: 298–303. 7924260 10.1037/0735-7036.108.3.298

[52] Gardner CR (1985) Distress vocalization in rat pups. A simple screening method for anxiolytic drugs. J Pharmacol Methods 14: 181–187. 2865408 10.1016/0160-5402(85)90031-2

[53] Miczek KA, Weerts EM, Vivian JA, Barros HM (1995) Aggression, anxiety and vocalizations in animals: GABAA and 5-HT anxiolytics. Psychopharmacology (Berl) 121: 38–56. 8539340 10.1007/BF02245590

[54] Miczek KA, Yap JJ, Covington HE 3rd (2008) Social stress, therapeutics and drug abuse: preclinical models of escalated and depressed intake. Pharmacol Ther 120: 102–128. 18789966 10.1016/j.pharmthera.2008.07.006 PMC2713609

[55] Morse WH, Skinner BF (1958) Some factors involved in the stimulus control of operant behavior. J Exp Anal Behav 1: 103–107. 14476352 10.1901/jeab.1958.1-103 PMC1403869

[56] Morris R (1984) Developments of a water-maze procedure for studying spatial learning in the rat. J Neurosci Methods 11: 47–60. 6471907 10.1016/0165-0270(84)90007-4

[57] Papageorgiou IE, Gabriel S, Fetani AF, Kann O, Heinemann U (2011) Redistribution of astrocytic glutamine synthetase in the hippocampus of chronic epileptic rats. Glia 59: 1706–1718. 21780187 10.1002/glia.21217

[58] Suarez I, Bodega G, Fernandez B (2002) Glutamine synthetase in brain: effect of ammonia. Neurochem Int 41: 123–142. 12020613 10.1016/s0197-0186(02)00033-5

[59] Martinez-Hernandez A, Bell KP, Norenberg MD (1977) Glutamine synthetase: glial localization in brain. Science 195: 1356–1358. 14400 10.1126/science.14400

[60] Hallermayer K, Harmening C, Hamprecht B (1981) Cellular localization and regulation of glutamine synthetase in primary cultures of brain cells from newborn mice. J Neurochem 37: 43–52. 6114136 10.1111/j.1471-4159.1981.tb05289.x

[61] Skolnick P, Popik P, Trullas R (2009) Glutamate-based antidepressants: 20 years on. Trends Pharmacol Sci 30: 563–569. 19837463 10.1016/j.tips.2009.09.002

[62] Hashimoto K (2010) Role of the mTOR signaling pathway in the rapid antidepressant action of ketamine. Expert Rev Neurother 11: 33–36. 10.1586/ern.10.176 21158553

[63] Welberg L (2010) Psychiatric disorders: Ketamine modifies mood through mTOR. Nat Rev Neurosci 11: 666. 10.1038/nrn2916 21080533

[64] Breier A, Malhotra AK, Pinals DA, Weisenfeld NI, Pickar D (1997) Association of ketamine-induced psychosis with focal activation of the prefrontal cortex in healthy volunteers. Am J Psychiatry 154: 805–811. 9167508 10.1176/ajp.154.6.805

[65] Rowland LM, Bustillo JR, Mullins PG, Jung RE, Lenroot R, et al (2005) Effects of ketamine on anterior cingulate glutamate metabolism in healthy humans: a 4-T proton MRS study. Am J Psychiatry 162: 394–396. 15677610 10.1176/appi.ajp.162.2.394

[66] Moghaddam B, Adams B, Verma A, Daly D (1997) Activation of glutamatergic neurotransmission by ketamine: a novel step in the pathway from NMDA receptor blockade to dopaminergic and cognitive disruptions associated with the prefrontal cortex. J Neurosci 17: 2921–2927. 9092613 10.1523/JNEUROSCI.17-08-02921.1997 PMC6573099

[67] Trullas R, Skolnick P (1990) Functional antagonists at the NMDA receptor complex exhibit antidepressant actions. Eur J Pharmacol 185: 1–10. 2171955 10.1016/0014-2999(90)90204-j

[68] Heresco-Levy U, Javitt DC, Gelfin Y, Gorelik E, Bar M, et al (2006) Controlled trial of D-cycloserine adjuvant therapy for treatment-resistant major depressive disorder. J Affect Disord 93: 239–243. 16677714 10.1016/j.jad.2006.03.004

[69] Kelly JP, Wrynn AS, Leonard BE (1997) The olfactory bulbectomized rat as a model of depression: an update. Pharmacol Ther 74: 299–316. 9352586 10.1016/s0163-7258(97)00004-1

[70] Adamec RE, Burton P, Shallow T, Budgell J (1999) Unilateral block of NMDA receptors in the amygdala prevents predator stress-induced lasting increases in anxiety-like behavior and unconditioned startle–effective hemisphere depends on the behavior. Physiol Behav 65: 739–751. 10073475 10.1016/s0031-9384(98)00225-x

[71] Heimer L (1968) Synaptic distribution of centripetal and centrifugal nerve fibres in the olfactory system of the rat. An experimental anatomical study. J Anat 103: 413–432. 4879162 PMC1231661

[72] Rex A, Voigt JP, Voits M, Fink H (1998) Pharmacological evaluation of a modified open-field test sensitive to anxiolytic drugs. Pharmacol Biochem Behav 59: 677–683. 9512071 10.1016/s0091-3057(97)00461-9

[73] Rodgers RJ, Boullier E, Chatzimichalaki P, Cooper GD, Shorten A (2002) Contrasting phenotypes of C57BL/6JOlaHsd, 129S2/SvHsd and 129/SvEv mice in two exploration-based tests of anxiety-related behaviour. Physiol Behav 77: 301–310. 12419406 10.1016/s0031-9384(02)00856-9

[74] Merali Z, Levac C, Anisman H (2003) Validation of a simple, ethologically relevant paradigm for assessing anxiety in mice. Biol Psychiatry 54: 552–565. 12946884 10.1016/s0006-3223(02)01827-9

[75] Minkeviciene R, Banerjee P, Tanila H (2008) Cognition-enhancing and anxiolytic effects of memantine. Neuropharmacology 54: 1079–1085. 18378262 10.1016/j.neuropharm.2008.02.014

[76] Winslow JT, Insel TR, Trullas R, Skolnick P (1990) Rat pup isolation calls are reduced by functional antagonists of the NMDA receptor complex. Eur J Pharmacol 190: 11–21. 1963846 10.1016/0014-2999(90)94107-9

[77] Santarelli L, Saxe M, Gross C, Surget A, Battaglia F, et al (2003) Requirement of hippocampal neurogenesis for the behavioral effects of antidepressants. Science 301: 805–809. 12907793 10.1126/science.1083328

[78] Mothet JP, Pollegioni L, Ouanounou G, Martineau M, Fossier P, et al (2005) Glutamate receptor activation triggers a calcium-dependent and SNARE protein-dependent release of the gliotransmitter D-serine. Proc Natl Acad Sci U S A 102: 5606–5611. 15800046 10.1073/pnas.0408483102 PMC556243

[79] Bado P, Madeira C, Vargas-Lopes C, Moulin TC, Wasilewska-Sampaio AP, et al (2011) Effects of low-dose D-serine on recognition and working memory in mice. Psychopharmacology (Berl) 218: 461–470. 21556803 10.1007/s00213-011-2330-4

[80] Hashimoto A, Chiba S (2004) Effect of systemic administration of D-serine on the levels of D- and L-serine in several brain areas and periphery of rat. Eur J Pharmacol 495: 153–158. 15249164 10.1016/j.ejphar.2004.05.036

[81] Zhang Z, Gong N, Wang W, Xu L, Xu TL (2008) Bell-shaped D-serine actions on hippocampal long-term depression and spatial memory retrieval. Cereb Cortex 18: 2391–2401. 18281302 10.1093/cercor/bhn008

[82] Shimazaki T, Kaku A, Chaki S (2010) D-Serine and a glycine transporter-1 inhibitor enhance social memory in rats. Psychopharmacology (Berl) 209: 263–270. 20198471 10.1007/s00213-010-1794-y

[83] Panizzutti R, Rausch M, Zurbrugg S, Baumann D, Beckmann N, et al (2005) The pharmacological stimulation of NMDA receptors via co-agonist site: an fMRI study in the rat brain. Neurosci Lett 380: 111–115. 15854761 10.1016/j.neulet.2005.01.062

[84] Platel JC, Gordon V, Heintz T, Bordey A (2009) GFAP-GFP neural progenitors are antigenically homogeneous and anchored in their enclosed mosaic niche. Glia 57: 66–78. 18661547 10.1002/glia.20735

